# Genetic landscape of 125 pharmacogenes in Chinese from the Chinese Millionome Database

**DOI:** 10.1038/s41598-021-98877-x

**Published:** 2021-09-28

**Authors:** Guangzhao Qi, Jingmin Zhang, Chao Han, Yubing Zhou, Duolu Li, Pengfei Ma

**Affiliations:** 1 Department of Pharmacy, The First Affiliated Hospital of Zhengzhou University, No. 1, Jianshe East Road, Zhengzhou, 450052 China; 2 Department of Hepatic Surgery, The First Affiliated Hospital of Zhengzhou University, Zhengzhou, China

**Keywords:** Clinical genetics, Medical genetics, Molecular medicine, Risk factors, Drug safety

## Abstract

Inter-individual differences of drug responses could be attributed to genetic variants of pharmacogenes such as cytochrome P450 (CYP), phase 2 enzymes, and transporters. In contrast to extensive studies on the genetic polymorphisms of CYP gene, genetic mutation spectrum of other pharmacogenes was under-representative in the pharmacogenetics investigations. Here we studied the genetic variations of 125 pharmacogenes including drug transporters, non-CYP phase 1 enzymes, phase 2 enzymes, nuclear receptors and others in Chinese from the Chinese Millionome Database (CMDB), of which 38,188 variants were identified. Computational analyses of the 2554 exonic variants found 617 deleterious missense variants, 91.1% of which were rare, and of the 54 loss-of-function (splice acceptor, splice donor, start lost, and stop gained) variants, 53 (98.1%) were rare. These results suggested an enrichment of rare variants in functional ones for pharmacogenes. Certain common functional variants including *NUDT15* 13:48611934 G/A (rs186364861), *UGT1A1* 2:234676872 C/T (rs34946978), and *ALDH2* 12:112241766 G/A (rs671) were population-specific for CMDB Chinese because they were absent (with a zero of variant allele frequency) or very rare in other gnomAD populations. These findings might be useful for the further pharmacogenomics research and clinical application in Chinese.

## Introduction

Increasing whole genome sequences data provide a goldmine for genetic studies on the disease etiology and drug therapeutics. Genetic variants of cytochrome P450 (CYP) genes performed an important role in the inter-individual differences of drug response^1^, and clinical implementation of several CYP variation-drug pairs such as CYP2C19*2–3 and clopidogrel, CYP2C9*2–3 and warfarin, and CYP3A5*3 and tacrolimus has been applied substantially worldwide^2,3^. Recently, pharmacogenes other than *CYP* such as *ABCC2* (ATP-binding cassette subfamily C member 2) transporters, *UGT2B7* (uridine-5'-diphosphate (UDP) glucuronosyltransferase family 2 member B7) and *F2R* (coagulation factor II (thrombin) receptor) were found to be of importance in the drug efficacy and adverse drug reactions^4–6^. However, information on the genetic variations of these pharmacogenes including non-CYP phase 1 enzymes, phase 2 enzymes, nuclear receptors and drug transporters in mainland Chinese was limited, to our knowledge. In addition, limited number of subjects in these studies was a common shortcoming, which weakened their power of application.

As the price of whole genome sequencing reduced to about $1000 dollars per genome, more genome sequencing projects across nations have been performed including the 1000 Genomes Project, the UK100K Genomes Project and the Genome Aggregation Database (gnomAD)^7,8^. Analysis of more than 130,000 whole genome sequences identified genetic variants with important clinical relevance in the ABC transporters, solute carrier (SLC) superfamily of transporters and organic anion transporting polypeptides (OATP) genes^9–11^. Mining of the genome sequence database could identify highly population-specific variants. For instance, SLC22A1*2-*5 involved in drug response of imatinib, metformin and opioids are absent in East Asians while own allele frequencies up to 21.9% in other world populations. Whereas, *SLC22A1* L160F is common in East Asians with a minor allele frequency of 0.142 but lowest in African population (minor allele frequency = 0.038)^10^. In addition, the ratios of population-specific variations differed among *ABC* genes from 70% in *ABCA7* to 92% in *ABCE1*, while only 0.3% of variations were shared among all seven world groups^11^. These results demonstrated that the need to better understand drug efficacy in participants of diverse ancestral backgrounds. However, these whole genome sequences database mainly comprised European populations while Chinese population were underrepresented.

A large-scale Chinese population sequencing project named the Chinese Millionome Database (CMDB) recently investigated 141,431 whole genome sequences from Chinese women who took a non-invasive prenatal testing^12^. Our previous study of 57 *CYP* genes and *POR* (cytochrome P450 oxidoreductase) in CMDB provided a comprehensive data set of P450 genes covering the whole mainland China provinces and 37 ethnicities^13^. In the present study we investigated the genetic variations of 125 pharmacogenes including non-CYP phase 1 enzymes, phase 2 enzymes, drug transporters, nuclear receptors and others in Chinese from the CMDB.

## Materials and methods

### Data sources

**Figure 1:**
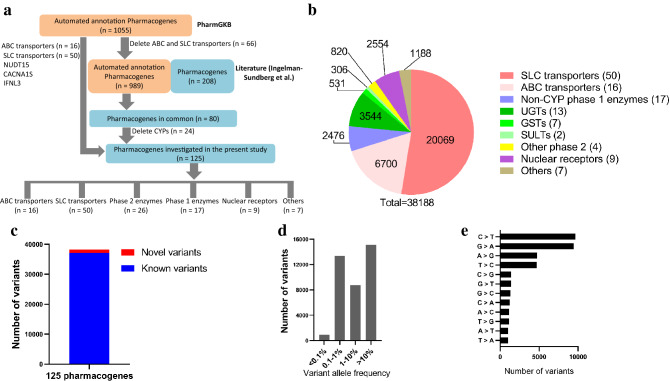
The genetic landscape of 125 pharmacogenes. (**a**) Flowchart of screen of the studied pharmacogenes. (**b**) Pie chart showing the distribution of the identified 38,188 variants among ABC transporters, SLC transporters, non-CYP phase 1 enzymes, phase 2 enzymes, nuclear receptors, and others. (**c**) 1038 (2.7%) of these 38,188 variants we identified were novel as compared to dbSNP release 135. (**d**) Number of variants with different variant allele frequencies. (**e**) Number of variants with different nucleotide mutation type.

One-hundred and twenty-five pharmacogenes, including 16 ABC transporters, 50 SLC transporters, 17 phase 1 enzymes, 26 phase 2 enzymes, 9 nuclear receptors and 7 others (including *ACE*, *CDA*, *POR*, *NUDT15*, *CACNA1S*, *G6PD*, *IFNL3*), were obtained through the pipeline in Fig. 1a. Briefly, the automated annotation text-mining system in PharmGKB (https://www.pharmgkb.org) was performed to scan sentences in literatures from PubMed. When the sentences included information linking a chemical and a variation, the text-mining system would annotate the sentence as pharmacogenomics information. And 1055 automated annotation pharmacogenes were identified (accessed 1 March, 2020), which were compared to pharmacogenes studied in reference (Ingelman- Sundberg et al.)^14^. Whole-genome sequencing data from 141,431 Chinese including 37 ethnicities living in 31 provinces of mainland China in CMDB were used to collect genetic variants information across the above 125 pharmacogenes (accessed 21 March, 2020). In brief, raw variants were gained using a *P* value less than 10^–6^ by the maximum likelihood model in the accessible genomic regions. Then, a Bayesian Gaussian mixture model was applied to assign every variant candidate a Phred-scaled probabilistic score (VQSR score) demonstrating the probability that this variant was a genuine polymorphic variant. The higher VQSR score indicated the higher probability that the variant candidate was a true polymorphic variation. High transition versus transversion (Ti/Tv) ratio is found for the raw call set (maximum 8.9 for novel variants and 3.4 for known variants) but it would decrease when the filtration threshold for VQSR score increases. A 35 of filtration threshold of VQSR score was applied indicating a Ti/Tv ratio of 2.4 for novel variants and of 2.2 for known variants^12^. Novel variant (without one rs number) was defined relative to dbSNP release 135. Variants with a variation allele frequency (VAF) less than 1% were defined as rare and variants with a VAF more than 1% were defined as common. The VAF in different gnomAD populations were collected from gnomAD browser (http://www.gnomad-sg.org/) in version 3.1.

### Variant effect prediction

The functional consequences of missense variants were predicted through a panel of online computational algorithms including SIFT (http://sift-dna.org), PolyPhen2 (http://genetics.bwh.harvard.edu/pph2/), and PROVEAN (http://provean.jcvi.org/index.php). Missense variation was categorized as deleterious when the ≥ 50% of the above algorithms predicted a damaging effect on the variation. In addition, splice acceptor and splice donor (in total of 8) were assessed by Combined Annotation Dependent Depletion (CADD) (https://cadd.gs.washington.edu/) to be deleterious, and start lost, and stop gained variants were regarded as putatively loss-of-function variants.

### Statistical analysis

Kruskal–Wallis test was utilized to compare the differences between variants number across pharmacogene subfamilies (GraphPad Prism version 8) (www.graphpad.com). Linear regression was used to analyze the relationship between missense, deleterious and total variants numbers and the corresponding gene length in different pharmacogenes groups. The chi-square test or Fisher’s exact test when needed was used for comparative analysis of the variant allele frequencies for the *ABCC4*, *SLCO1B1*, *ALDH2*, *TPMT*, *UGT1A1*, *VDR* and *NUDT15* polymorphisms between different populations in gnomAD and CMDB Chinese using R version 4.1.0 (https://www.r-project.org/). *P* < 0.05 was recognized as statistically significant, while the Bonferroni Correction was used to adjust the significance threshold (*P* < 0.002 (0.05/18)) for the multiple testing of the linear regression analysis between the number of total variants, missense variants and deleterious variants and the corresponding gene length among the 6 pharmacogenes groups, and to adjust the significance threshold (*P* < 0.0006 (0.05/72)) for the multiple testing of the comparison for variants allele frequencies between CMDB Chinese and diverse gnomAD populations.

## Results

### Genetic variability overview in 125 human pharmacogenes

We analyzed the genetic variations in 125 genes with importance for drug response utilizing whole genome sequencing data from 141,431 unrelated Chinese subjects. Totally, we identified 38,188 variants distributed across transporter genes (26,769 variants in 66 genes), genes encoding phase 1 (2476 variants in 17 genes) and phase 2 enzymes (5201 variants in 26 genes), nuclear receptors (2554 variants in 9 genes), and other pharmacogenes with diverse functions (1188 variants in 7 genes; Fig. 1b). In addition, 1038 (2.7%) of these 38,188 variants identified were novel as compared to dbSNP release 135 (Fig. 1c). Notably, 14,294 (37.4%) variants were rare with a VAF less than 1% (Fig. 1d). Of the twelve nucleotide transversions and transitions, C > T (25.3%) and G > A (24.8%) constituted the most common types of mutations, representing over half of the mutated nucleotides in 125 pharmacogenes (Fig. 1e).

**Figure 2:**
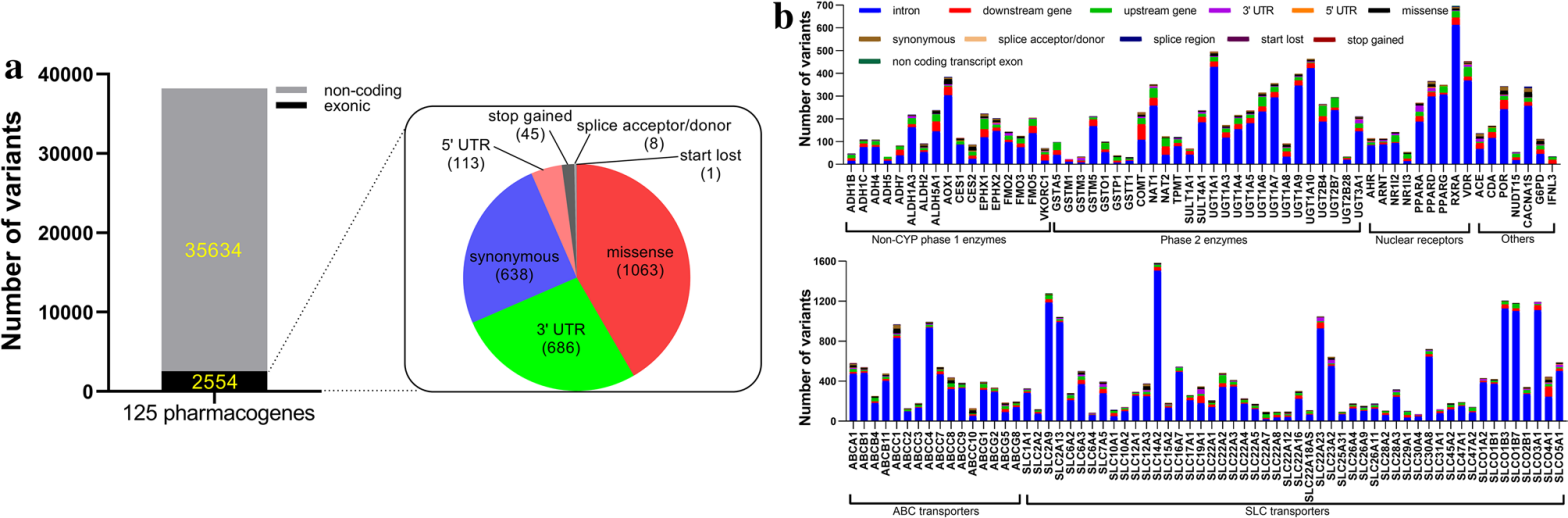
Gene region pattern of 125 pharmacogenes. (**a**) Of these 38,188 variants, 35,634 variants were non-coding ones (intronic, upstream and downstream regions) while 2554 (6.7%) were exonic variants. Pie chart showing the distribution of the identified 2554 exonic variants. (**b**) Number of variants with different gene region distributions in each of ABC transporters, SLC transporters, non-CYP phase 1 enzymes, phase 2 enzymes, nuclear receptors, and others pharmacogenes.

Of these 38,188 variants, 35,634 variants were non-coding ones (intronic, upstream and downstream regions) while 2554 (6.7%) were exonic variants (Fig. 2a). The majority of 2554 exonic variants were missense (n = 1063; 41.6%), followed by untranslated region (UTR) (n = 799; 31.3%) and synonymous (n = 638; 25.0%; Fig. 2a). The most variants in ABC transporters, SLC transporters, non-CYP phase 1 enzymes, phase 2 enzymes, nuclear receptors, and others were observed in *ABCC4* (n = 993), *SLC14A2* (n = 1582), *AOX1* (n = 384), *UGT1A1* (n = 495), *RXRA* (n = 696), and *POR* (n = 343), respectively (Fig. 2b). The least variants in ABC transporters, SLC transporters, non-CYP phase 1 enzymes, phase 2 enzymes, nuclear receptors, and others were observed in *ABCC2* (n = 126), *SLC30A4* (n = 71), *ADH5* (n = 32), *GSTM1* (n = 22), *NR1I3* (n = 54), and *IFNL3* (n = 34), respectively. Intronic variants were the most type across the 125 pharmacogenes (Fig. 2b).

### The majority of variants with putative clinical relevance were rare

**Figure 3:**
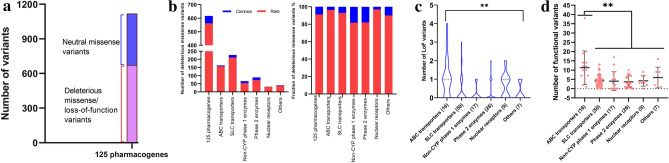
Rare variants were the majority of putatively functional pharmacogenes variants. (**a**) Number of neutral missense variants were 446 while deleterious missense and loss-of-function variants were 671 for the studied 125 pharmacogenes. (**b**) Number and fraction of deleterious missense variants with different variant allele frequencies in diverse pharmacogenes families. (**c**) Number of loss-of-function variants in different pharmacogenes families. (**d**) Number of functional variants (deleterious missense plus loss-of-function variants) in different pharmacogenes families.

**Figure 4:**
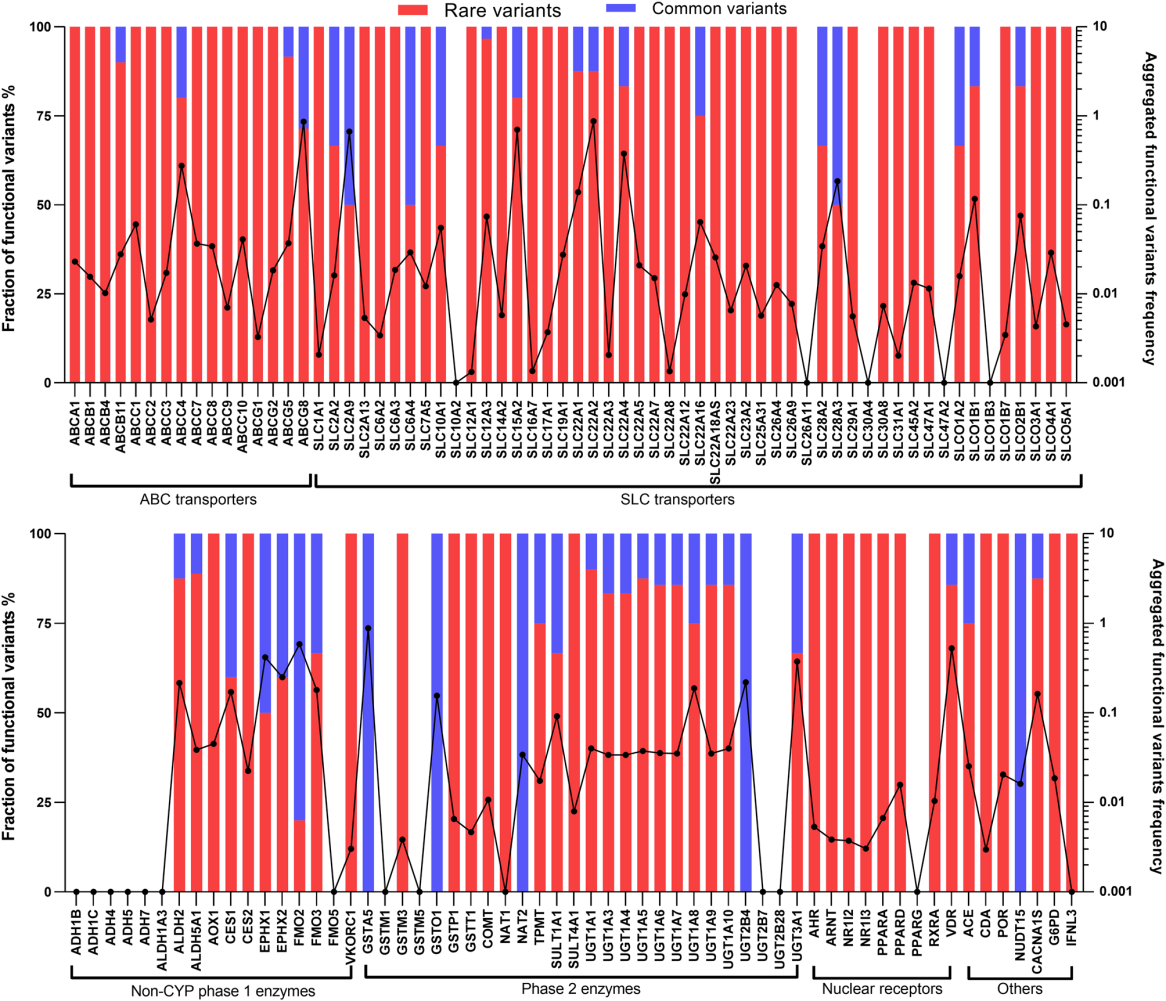
Frequency distribution of functional variants in individual pharmacogene. The aggregated putatively functional variants frequency of each of ABC transporters, SLC transporters, non-CYP phase 1 enzymes, phase 2 enzymes, nuclear receptors, and others pharmacogenes is plotted in log scale and indicated as dots connected by the black line (right y-axis). The fraction of the functional variation that is allotted to common (blue) or rare (red) variants is indicated on the left y-axis.

Neutral missense variants were comprised of 446 while deleterious missense and loss-of-function variants were comprised of 671 (see the “Methods” section for details; Fig. 3a). Of the 617 deleterious missense variants, 562 (91.1%) were rare while only 55 (8.9%) were common (Fig. 3b), which suggested an enrichment of rare missense variants in functional ones for pharmacogenes. The number and percentage of rare deleterious missense variants in ABC transporters, SLC transporters, non-CYP phase 1 enzymes, phase 2 enzymes, nuclear receptors, and others were 157 (96.3%), 211 (93.0%), 54 (81.8%), 73 (82.0%), 31 (96.9%), and 36 (90.0%), respectively. Of the 54 loss-of-function (splice acceptor, splice donor, start lost, and stop gained) variants, 53 (98.1%) were rare, except one common splice donor variant in *SULT1A1* (16:28,631,383 C/G; rs79527462; c.-266 + 1 N > C) with a VAF of 8.71%. A significant different distribution of loss-of-function variants among ABC transporters (n = 18), SLC transporters (n = 18), non-CYP phase 1 enzymes (n = 3), phase 2 enzymes (n = 7), nuclear receptors (n = 6), and others (n = 2) were observed (Fig. 3c; *P* = 0.0047). Totally, the average distribution of functional variants (deleterious missense plus loss-of-function variants) in ABC transporters (mean ± SD: 11.2 ± 9.03) was significantly more than those in SLC transporters (4.2 ± 3.22), non-CYP phase 1 enzymes (4.1 ± 5.09), phase 2 enzymes (3.7 ± 3.15), nuclear receptors (4.1 ± 2.86), and others (5.9 ± 5.60) (Fig. 3d; *P* = 0.0048). However, we re-test the relationship between pharmacogenes family and loss-of-function burden and functional variants adjusting for gene size and no significant difference was found among the pharmacogenes families (*P* = 0.0916 for LoF variants and *P* = 0.1212 for functional variants). The significantly larger average distribution of functional variants in *ABC* transporters than those in other groups could be attributed to the gene size and the large number of exons across the *ABC* superfamily relative to the other genes/gene families tested. To evaluate the functional importance of rare variants in individual pharmacogene, we calculated the percentage of rare and common functional variants (deleterious missense plus loss-of-function variants) and aggregated frequencies of splice acceptor, splice donor, start lost, stop gained and putatively deleterious missense variants in each of the 125 studied pharmacogenes (Fig. 4). In total, we could observe a substantially different distribution and pattern of genetic diversity among the 125 pharmacogenes studied. No functional variants with a zero of aggregated functional variants frequency were identified in pharmacogenes including *SLC10A2*, *SLC26A11*, *SLC30A4*, *SLC47A2*, *SLCO1B3*, *ADH1B*, *ADH1C*, *ADH4*, *ADH5*, *ADH7*, *ALDH1A3*, *FMO5*, *GSTM1*, *GSTM5*, *UGT2B7*, *UGT2B28*, and *PPARG*. Several common variants constituted the majority (common variants fraction ≥ 50%) of genetic variability with functional importance for such pharmacogenes as *SLC2A9*, *SLC6A4*, *SLC28A3*, *EPHX1*, *FMO2*, *GSTA5*, *GSTO1*, *NAT2*, *UGT2B4*, and *NUDT15*, with an aggregated functional variants frequency of 66.56%, 2.93%, 18.42%, 41.67%, 58.59%, 88.17%, 15.53%, 3.40%, 21.90%, 1.61%, respectively. Whereas, the remaining 98 (78.4%) pharmacogenes including *ABCB1*, *SLC10A1*, *SLCO1B1*, *ALDH2*, *GSTP1*, *TPMT*, *SULT1A1*, *UGT1A1*, *AHR*, *G6PD*, and *IFNL3*, the majority of the functionality of which was governed by rare genetic variations (rare variants fraction ≥ 50%; Fig. 4). The most highly polymorphic pharmacogenes were *GSTA5* (aggregated functional variants frequency 88.17%), *SLC22A2* (87.33%), *ABCG8* (86.09%), *SLC15A2* (69.86%), *SLC2A9* (66.56%), *FMO2* (58.59%), and *VDR* (52.47%), whereas the least numbers of functional variants were identified for *SLC16A7* (0.14%), *SLC22A8* (0.13%), *SLC12A1* (0.13%), *NAT1* (0.10%), and *IFNL3* (0.09%). The fold between the highest frequency of genetic variants predicted to impact the function of the gene product (*GSTA5* 88.17%) and lowest (*IFNL3*, 0.09%) was approximately 979.7. Accordingly, overall genetic variation as well as the fraction of functional variants that was allotted to rare variants differs significantly among the 125 studied pharmacogenes.

### Different correlation patterns between number of variants and gene length among pharmacogene groups

**Figure 5:**
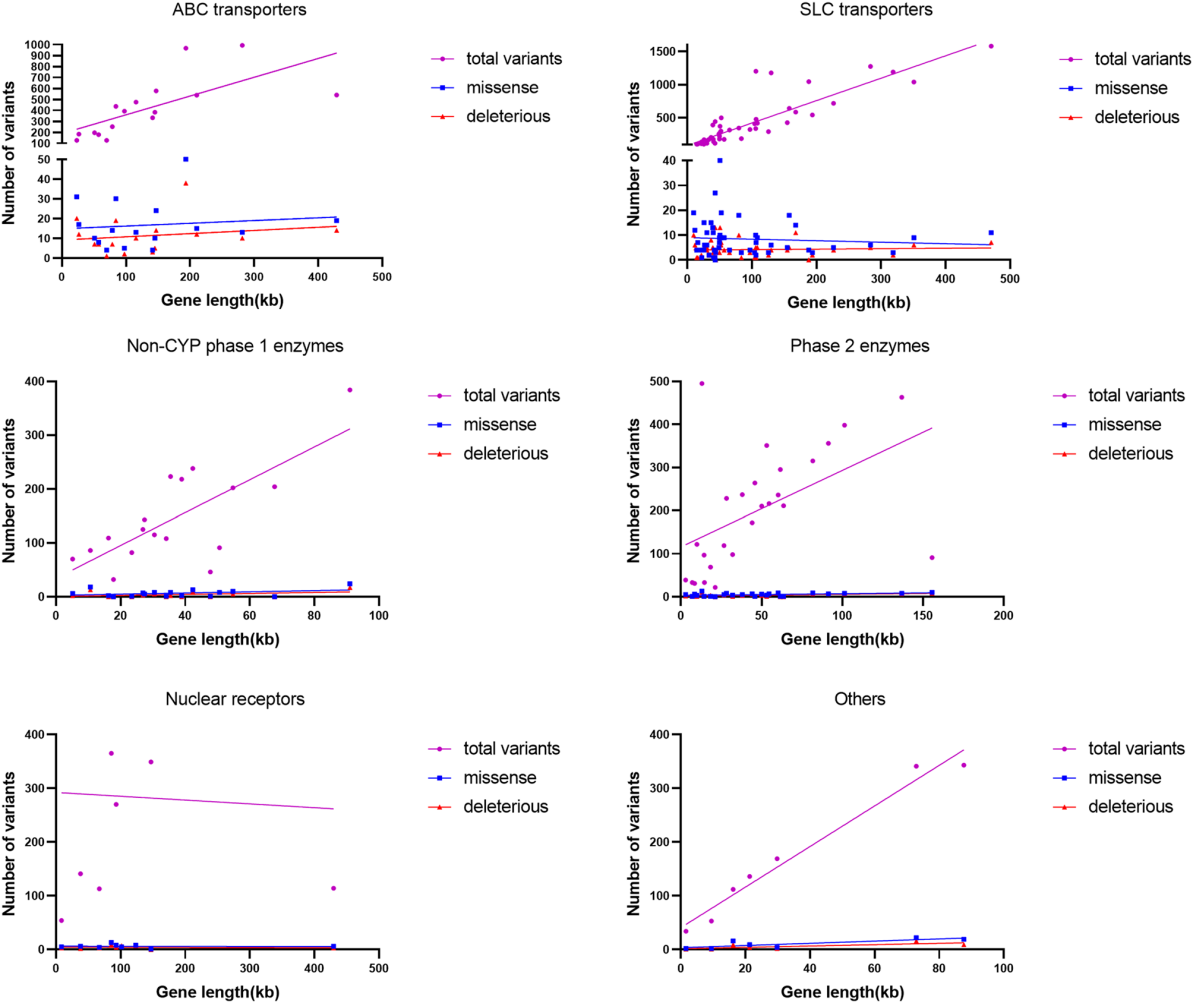
Correlation of variants number with the gene length. The relationship between the number of total variants, missense variants and deleterious variants and the corresponding gene length among the 6 pharmacogenes groups were different.

Through linear regression analysis, we investigated the relationship between the number of total variants, missense variants and deleterious variants and the corresponding gene length among the 6 pharmacogenes groups. After the Bonferroni Correction for the multiple test, no significant correlation between the number of total variants (*P* = 0.0083), missense variants (*P* = 0.0234), and deleterious variants (*P* = 0.0081) and the corresponding gene length was found in phase 2 enzymes group (Fig. 5). Moreover, no correlation between the number of total variants (*P* = 0.9141), missense variants (*P* = 0.8818), and deleterious variants (*P* = 0.7784) and the corresponding gene length was found in nuclear receptors group. The trend upwards between the nuclear receptor total variant category and gene length was damaged by a single outlier (*AHR*, aryl hydrocarbon receptor). *AHR*, a member of the basic helix-loop-helix-period-aryl hydrocarbon receptor nuclear translocator-single-minded (bHLH-PAS) family of transcription factors, has a gene length of 429.79 kb but only 114 variants in total, which might be attributed to its conservative role as the biological sensor in initiating gene expression procedures in responses to exogenous and endogenous signals. In addition, the relationship between the number of total variants and gene length was found to be significant in SLC transporters (*P* < 0.0001), and non-CYP phase 1 enzymes (*P* = 0.0007) respectively, but not significant in ABC transporters (*P* = 0.0038). Furthermore, no significant correlation between the number of missense and deleterious variants and gene length was found in the three pharmacogenes groups (all *P* > 0.05). The others pharmacogenes group (totally 7 genes) had the significant correlation between the number of total variants (*P* < 0.0001) and gene length, while the relationship between the number of deleterious variants (*P* = 0.0559) and missense variants (*P* = 0.0299) and the corresponding gene length in this group were not significant.

### Different VAF with clinical relevance among CMDB and gnomAD populations

To demonstrate the VAF in CMDB Chinese and other populations, we chose the common functional variants in *ABCC4*, *SLCO1B1*, *ALDH2*, *TPMT*, *UGT1A1*, *VDR* and *NUDT15* from the 6 pharmacogenes groups. Diverse variant allele frequencies of the chosen 8 variants in different gnomAD populations (African, Ashkenazi Jewish, Amish, Middle Eastern, Latino, European (non-Finnish), European (Finnish), East Asian, and South Asian) and CMDB Chinese were found as shown in Table 1. The *P* values are shown in Supplementary Table S1. Especially some common functional variants were population-specific for CMDB Chinese because they were absent (with a zero of VAF) or very rare in other gnomAD populations, including *NUDT15* 13:48611934 G/A (rs186364861), *UGT1A1* 2:234676872 C/T (rs34946978), and *ALDH2* 12:112241766 G/A (rs671). These genetic polymorphisms in the pharmacogenes in different populations might impact the drug response in the kinds of ethnicities.

**Table 1:** VAF with clinical relevance in CMDB Chinese and diverse gnomAD populations.

Gene	Variant	Consequence	Drugs involved	African	Ashkenazi Jewish	Amish	Middle Eastern	gnomAD_VAF					CMDB_VAF
								Latino	European (non-Finnish)	European (Finnish)	East Asian	South Asian	Chinese
ABCC4	13:95,859,035 C/A (rs2274407)	p.Lys304Asn	Methotrexate, Mercaptopurine, Zidovudine, Dipyridamole	0.167	0.1325	0.01429*	0.121	0.08122*	0.06825*	0.06309*	0.1759	0.1456	0.126
	13:95,863,008 C/A (rs11568658)	p.Gly187Trp		0.004779*	0.0415*	0.1404	0.05063*	0.08711*	0.02346*	0.04566*	0.1146	0.05763*	0.1155
SLCO1B1	12:21,331,549 T/C (rs4149056)	p.Val174Ala	Pravastatin, Rifampicin, Gemfibrozil	0.03168*	0.1771	0.06798*	0.2057	0.13	0.1587	0.2178	0.1279	0.04888*	0.1085
ALDH2	12:112,241,766 G/A (rs671)	p.Glu504Lys	Disulfiram, Ethanol	0.0001931*	0*	0*	0*	0.0009178*	0.0000294*	0.00009436*	0.2247	0.0008292*	0.2021
TPMT	6:18,130,918 T C (rs1142345)	p.Tyr240Cys	Azathioprine, Mercaptopurine , Cefazolin, Olsalazine	0.05485	0.01902	0.0165	0.02532	0.05058	0.04227	0.0293	0.01366	0.01863	0.01291
UGT1A1	2:234,676,872 C/T (rs34946978)	p.Pro364Leu	Irinotecan, Nilotinib, Atazanavir, Carvedilol	0.0002171*	0*	0*	0	0.001767*	0.0000294*	0*	0.01196*	0.001242*	0.01986
VDR	12:48,272,895 A/G (rs2228570)	p.Met51Thr	Calcipotriol, Calcitriol,Ergocalciferol	0.7799	0.5716	0.7368	0.7025	0.5778	0.6162	0.644	0.5698	0.7387	0.5141
NUDT15	13:48,611,934 G/A (rs186364861)	p.Val18Ile	Azathioprine, Mercaptopurine , Thioguanine, Magnesium	0*	0.000288*	0*	0	0*	0.0000147*	0*	0.01121	0.0008271*	0.01607

*VAF* variant allele frequency, *CMDB* Chinese Millionome Database, *gnomAD* Genome Aggregation Database, *ABCC4* ATP-Binding Cassette Subfamily C Member 4, *SLCO1B1* Solute Carrier Organic Anion Transporter Family Member 1B1, *ALDH2* Aldehyde Dehydrogenase 2 Family Member, *TPMT* Thiopurine S-Methyltransferase, *UGT1A1* UDP Glucuronosyltransferase Family 1 Member A1, *VDR* Vitamin D Receptor, *NUDT15* Nudix Hydrolase 15.

*P < 0.0006 compared with CMDB Chinese by chi-square test or Fisher’s exact test when needed.

## Discussion

The present study provided a comprehensive and systematic genetic overview of 125 pharmacogenes including 16 ABC transporters, 50 SLC transporters, 17 non-CYP phase 1 enzymes, 26 phase 2 enzymes, 9 nuclear receptors and 7 others in Chinese from 141,431 whole genome sequences preserved in CMDB. To the best of our knowledge, this is the first study on the genetic variation of 125 pharmacogenes in such a large-scale population in mainland Chinese. Europeans were over-representative in genomes sequencing projects such as gnomAD worldwide, whereas other populations including Chinese were under-representative in these projects. Therefore, it was not appropriate to use the results from these projects directly on other populations. Moreover, drug development trials neglected to study participants of diverse ancestries would result in poor generalizability of marketed drug efficacy information. Additionally, the withdrawal of marketed drugs due to adverse drug reactions might be attributed to the patients with specific genetic variation, while in fact they would be efficacious for other patients without such certain genetic variant. For example, functional alleles SLCO1A2*2 and SLCO1A2*3 involved in drug uptake of opioid receptor agonist deltorphin II and methotrexate were prevalent in Ashkenazim (VAF = 0.145 and 0.034, respectively) and Europeans (VAF = 0.136 and 0.062, respectively) while absent in East Asians (both VAF < 0.001)^9^. And our present study found some common functional pharmacogene variants such as *NUDT15* rs186364861, *UGT1A1* rs34946978 and *ALDH2* rs671 in CMDB Chinese were population-specific but absent in other populations (Table 1). These results were of key importance in designing the pharmacogenomics testing panel in Chinese. FDA (Food and Drug Administration) had provided 457 drug-gene pairs in the table of pharmacogenomics biomarkers in drug labeling (https://www.fda.gov/drugs/science-and-research-drugs/table-pharmacogenomic-biomarkers-drug-labeling), and before the whole genome sequencing becomes the routine testing in clinics, a cost-effective pharmacogenomics testing panel covering an individualized pharmacogenes variants testing items would be needed^15^.

The limitation of our present study is that lacking of haplotype information across pharmacogenes which is a main source of actionable variation for translating these data to clinically meaningful conclusions. According to a review by the Human Genetic Resources Administration of China (HGRAC), the individual genetic data from CMDB is unavailable^12^. We could obtain detailed summaries of the data such as allele frequencies and GWAS summary statistics. Unfortunately, ethnicity-specific allele frequencies across pharmacogenes are unavailable. However, the minorities in mainland China such as Mongolian, Tibetan and Uyghur populations owned different alleles and genotypes frequencies of pharmacogenes from those in Han Chinese^16^. Therefore, more detailed pharmacogenomic studies in these minorities would be needed to perform in future.

Genetic variability of the well-known pharmacogenes alleles could explain only part of the inter-individual differences in drug responses, and rare genetic variants in pharmacogenes such as *SLC30A8* could modulate antidepressant (desipramine or fluoxetine) treatment^17^. The potential role on the disease development and drug efficacy of the rare variants in these genetically polymorphic pharmacogenes would be interesting and needed to be studied further^18,19^. The present study demonstrated that 91.1% of the 617 deleterious missense variants in the 125 pharmacogenes were rare (Fig. 3b), which suggested the necessity to focus on the rare pharmacogenes variants in studying inter-individual variability in drug response. Analysis of 25 clinically relevant pharmacogenes in 291 genomes of the Thai population identified 121 putatively functional variants, majority of which were rare and specific to the Thais but absent from gnomAD database^20^. As more and more rare pharmacogene variants were found, the method to interpret their clinical implication were needed to be developed urgently^21,22^. Therefore, the database comprised of both the pharmacogenes genotype and drug response phenotype information should be constructed across the world.

Most of the drugs are metabolized by several enzymes, and often a combination of CYPs and non-CYPs. Although the majority of drug biotransformation is performed by the CYP enzymes, other pharmacogenes such as drug transporters and nuclear receptors might participate in many clinically important drugs^23,24^. Constitutive androstane receptor (CAR) rs2502815 polymorphism and the carbamazepine response in epilepsy patients was potentially relevant. However, our present study found the rare variants in CAR (NR1I3) formed the 100% fraction of the functional variation and the aggregated putatively functional variants frequency of CAR was 0.00305. These results might explain the minor role played by the non-CYP pharmacogenes in the pharmacogenetics studies, where functional alleles in these pharmacogenes were not considered because of the very low VAF in the studied populations. Twin studies on the pharmacokinetics of metoprolol and torsemide suggested that up to 90% of the variation in their pharmacokinetic parameters could be allotted to the subjects’ genetic makeup, whereas the known genetic variations of *CYP2D6*, *CYP2C9*, and *SLCO1B1* explained only 39%, 2%, and 39% of the pharmacokinetics variability, respectively^25^. These findings combined with our present results indicated that a substantial fraction of the heritable variation in the drug responses of clinically important medicines remained to be elucidated.

In conclusion, we comprehensively mapped the genetic landscape of 125 pharmacogenes in mainland Chinese from CMDB and identified 38,188 variants. Computational analyses of the 2554 exonic variants identified 617 deleterious missense variants, 91.1% of which were rare, and of the 54 loss-of-function (splice acceptor, splice donor, start lost, and stop gained) variants, 53 (98.1%) were rare. These results suggested an enrichment of rare variants in functional ones for pharmacogenes. Certain common functional variants including *NUDT15* 13:48611934 G/A (rs186364861), *UGT1A1* 2:234676872 C/T (rs34946978), and *ALDH2* 12:112241766 G/A (rs671) were population-specific for CMDB Chinese because they were absent (with a zero of VAF) or very rare in other gnomAD populations. These findings might be useful for the further pharmacogenomics research and clinical application in Chinese.

## Supplementary Information

Supplementary Information.
